# Novel Case of a Molecularly Confirmed Low-Grade Fibromyxoid Sarcoma of the Floor of the Mouth and Literature Review

**DOI:** 10.1007/s12105-026-01888-1

**Published:** 2026-01-30

**Authors:** Eugene G. Bestman, John K. Brooks, Scott D. Nelson, Ahmed S. Sultan, Njood Hawari, Samantha L. Jeffrey, Jettie Uyanne, Felix Kyle Yip, Kevin Artis, Manando Nakasaki

**Affiliations:** 1 Department of Oral and Maxillofacial Surgery, Harbor-UCLA Medical Center, 1000 W Carson Street, Torrance, Los Angeles, CA 90502 USA; 2 Department of Oncology and Diagnostic Sciences, University of Maryland School of Dentistry, Baltimore, MD USA; 3 Department of Pathology and Laboratory Medicine, David Geffen School of Medicine, University of California, Los Angeles, CA USA; 4 University of Maryland Marlene and Stewart Greenebaum Comprehensive Cancer Center, Baltimore, MD USA; 5 Department of Oncology and Diagnostic Sciences, Oral and Maxillofacial Pathology, University of Maryland School of Dentistry, Baltimore, MD USA; 6 Sarcoma Oncology Research Center, Cancer Center of Southern California, Santa Monica, CA USA

**Keywords:** Floor of the mouth, *FUS::CREB3L2* fusion, Low-grade fibromyxoid sarcoma, MUC4

## Abstract

**Background:**

Low-grade fibromyxoid sarcoma (LGFMS) is a deceptively bland spindle cell neoplasm with malignant potential, most commonly arising in the extremities and trunk, and limited to at least 110 cases in the head and neck. This report describes the first molecularly confirmed LGFMS of the floor of the mouth demonstrating a *FUS::CREB3L2* gene fusion.

**Case Presentation:**

An 18-year-old male presented with a painless floor of mouth mass of 1-year duration that was clinically suspected to represent a ranula. The mass was subjected to excisional biopsy. Histological examination was salient for an infiltrating proliferation of bland spindle cells arranged in whorled patterns within a collagenous stroma with focal myxoid change.

**Results:**

Immunohistochemical analysis revealed diffuse MUC4 expression, narrowing the diagnosis. Targeted RNA sequencing identified a *FUS::CREB3L2 *fusion involving *FUS exon 6 and CREB3L2 exon 5*, confirming LGFMS. At 1-year follow-up, the patient showed no clinical evidence of disease and no detectable circulating tumor DNA.

**Conclusion:**

This case highlights the critical role of molecular testing in the diagnosis of LGFMS and emphasizes the importance of appropriate management strategies, including aggressive local control and long-term surveillance, given the tumor’s risk of late recurrence and distant metastasis.

**Supplementary Information:**

The online version contains supplementary material available at 10.1007/s12105-026-01888-1.

## Introduction

Low-grade fibromyxoid sarcoma (LGFMS), also referred to as Evans tumor, was first reported in 1987 and is considered a rare malignant fibroblastic neoplasm with an estimated incidence of 0.18 per million [1]. Further, a search of a large tumor registry revealed that LGFMS represented 0.44% (33/7500) of total sarcoma accessions [2]. Some authors have contended that the actual number of LGFMS cases has been difficult to determine due to earlier challenges in rendering a confirmed molecular diagnosis and underreporting [3]. LGFMS typically affects individuals in their 20 s to 40 s, with a slight male predilection. Lesions usually present as a painless, slow-growing, and well-circumscribed deep-seated soft tissue mass that range in size from 2 to 26 cm (median- 4.5 cm) [1]. Some lesions may present superficially, confined to the dermis and/or subcutaneous tissue without involvement of the deep fascia or underlying skeletal muscle, and reported across a range of age groups, including pediatric and adult patients [4].

The most common sites of LGFMS include the trunk and proximal extremities [5–10]. The head and neck region is an atypical site for this tumor, with at least 135 published cases occurring within this region in the English language literature, the preponderance not providing cytogenetic findings [Supplemental file]. The objective of this report is to expand the knowledge of the presentation of LGFMS by describing the novel occurrence of this tumor in the floor of the mouth, initially suspected to be a ranula, and molecularly confirmed with *FUS::CREB3L2* fusion. A summary of affected patients with LGFMS of the head and neck with cytogenetic studies has also been provided.

## Case Report

An 18-year-old male was referred to the Oral and Maxillofacial Surgery Clinic at the Martin Luther King Jr. Outpatient Hospital (Willowbrook, California, USA) for evaluation of an asymptomatic mass of the anterior floor of the mouth, described by the patient as a “ball behind my tooth.” The patient denied dysgeusia, and had not experienced pain, paresthesia, or trauma within the affected region. He recalled that the lesion had been present for approximately 1 year, without fluctuation in size. The patient’s medical history and review of systems were unremarkable, and he was not taking any medications. He denied tobacco, alcohol, or illicit drug use, and his family history was noncontributory.

**Fig. 1:**
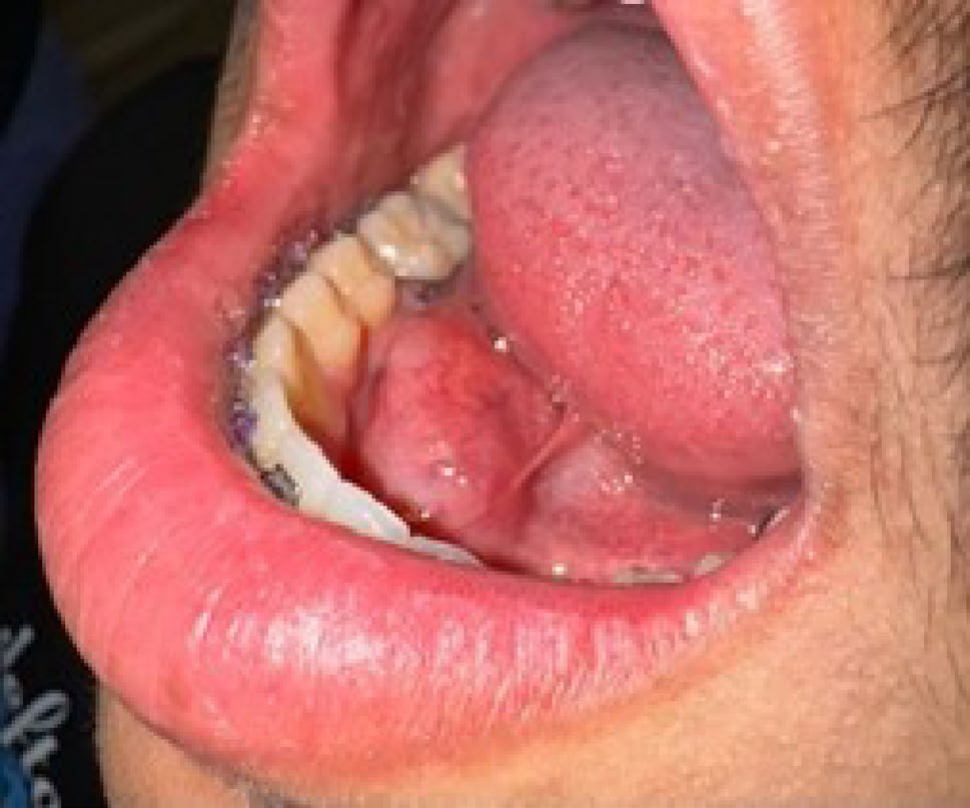
Clinical presentation of low-grade fibromyxoid sarcoma (LGFMS) of the floor of the mouth

**Fig. 2:**
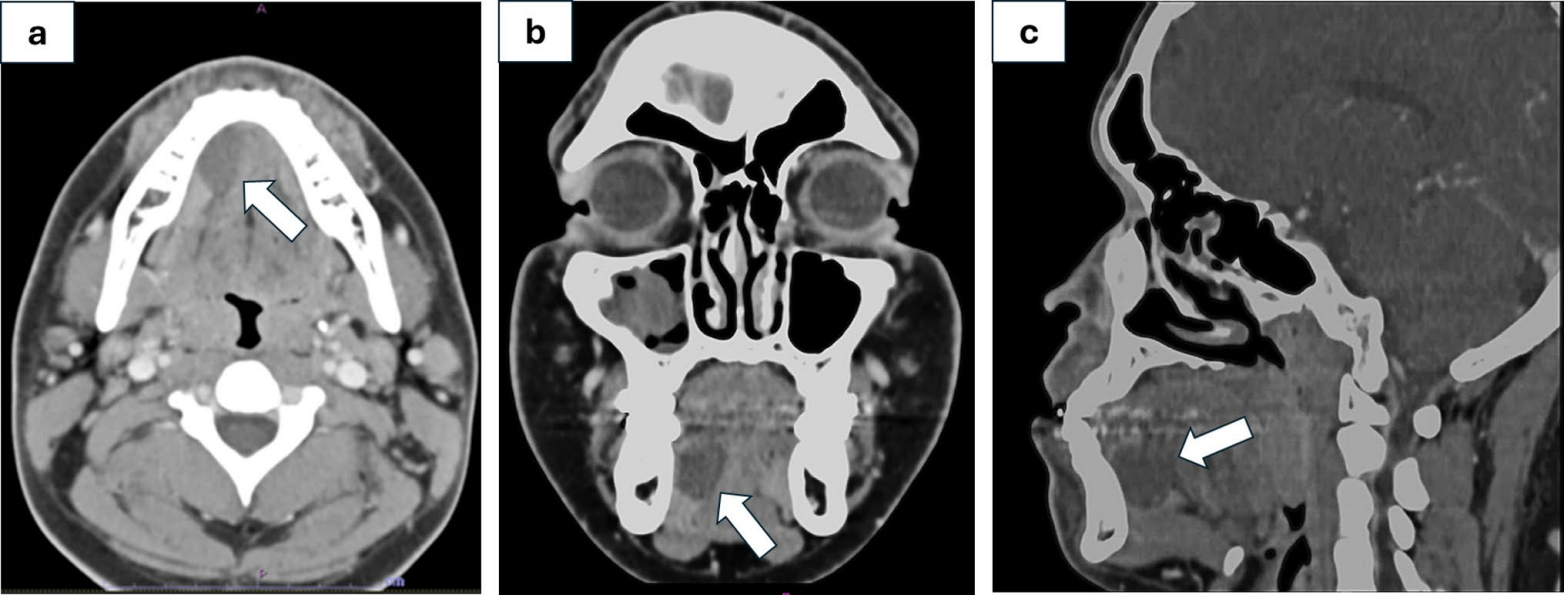
Computed tomography scan images show cystic mass appearing in the right anterior sublingual space (with arrow). **a** Coronal view. **b** Sagittal view. **c** Axial view

**Fig. 3:**
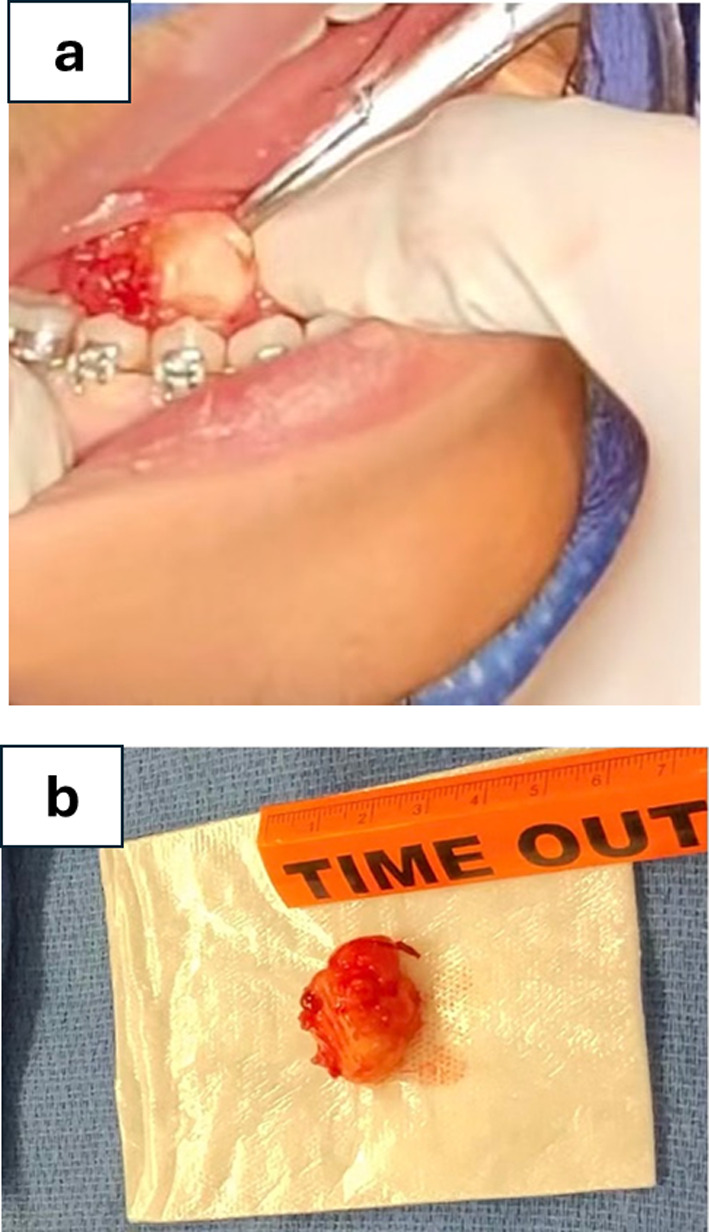
Operative views. **a** Capsulated lesion. **b** Surgical specimen

The extraoral examination was unremarkable. Intraoral examination revealed a 2 × 2 × 2 cm, raised, soft and mobile depressible swelling with normal color, of the right anterior floor of the mouth (Fig. 1). The clinical impression of the lesion was a ranula. Computed tomography (CT) examination demonstrated a 2.2 × 1.7 × 1.2 cm soft tissue density-based structure in the right anterior sublingual space (Fig. 2a–c). Preoperative laboratory studies were normal. Linear mucosal incision was made with a bovie electrocautery over the lesion. Blunt dissection was performed with Kelly forceps between mucosa, and the lesion was bluntly dissected to the level of the sublingual gland and completely excised (Fig. 3a, b).

**Fig. 4:**
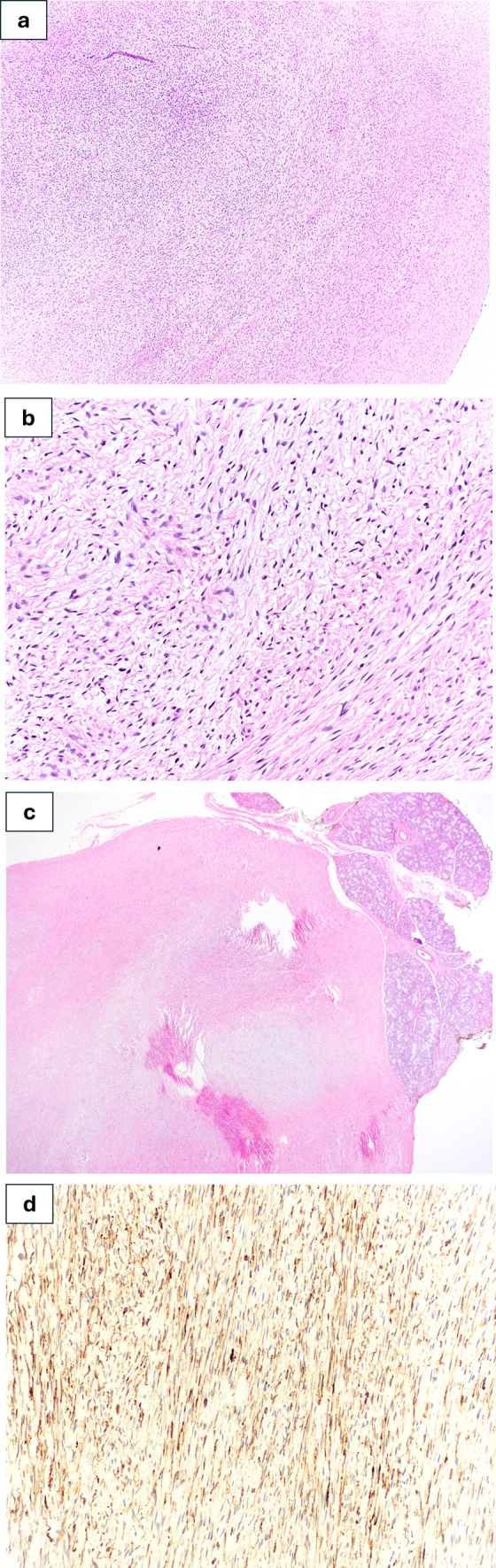
Photomicrographs show representative findings of LGFMS. **a** Moderately cellular spindle cell proliferation arranged in short fascicles within a collagenous stroma containing fine curvilinear blood vessels and focal myxoid transitional areas (hematoxylin and eosin staining, original magnification 40x). **b** Bland appearing spindle cells with mild nuclear pleomorphism (hematoxylin and eosin staining, original magnification 200×). **c** Focal areas of normal salivary glands adjacent to the tumor (hematoxylin and eosin staining, original magnification 20×). **d** Immunohistochemical staining was diffusely positive for MUC4 in tumor cells (original magnification 200×)

Histopathologic examination revealed an infiltrative proliferation of bland spindle cells within a collagenous stroma containing fine, curvilinear blood vessels and focal myxoid areas (Fig. 4a). The spindled cells exhibited small fusiform nuclei with mild nuclear pleomorphism in an arrangement of short fascicles and whirling growth patterns (Fig. 4b). The tumor appeared to be marginally excised and displayed focal areas of normal salivary glands (Fig. 4c). Immunohistochemical studies, performed at UCLA Medical center clinical and pathology laboratories using validated antibodies and standard staining protocols, stained diffusely positive for MUC4, using clone EP256 rabbit monoclonal antibody; dilution 1:25 (Bio SB Inc, Goleta, California). (Fig. 4d) and was negative for CD34, S100, SOX10, desmin, caldesmon, and beta-catenin, which supported diagnosis of LGFMS. RNA sequencing was performed using a targeted solid tumor fusion panel, which detected *FUS::CREB3L2* gene fusion.

The patient was then referred to the Sarcoma Oncology Center (Santa Monica, California, USA) for evaluation and management. Tissue samples were sent to Caris Life Sciences (Irving, Texas, USA) for RNA sequencing with a targeted solid tumor fusion panel, using Archer's AMP™ technology to interrogate specific regions of genes and featured a highly optimized oligonucleotide probe pool with specificity for the recurrent break points in the fusion partner genes that are recurrently rearranged in a variety of solid tumors. This panel evaluated regions of interest within 157 clinical genes for potential rearrangements. Sequencing of fusion libraries was performed on Illumina MiSeq Sequencing System (Illumina ISC, Baltimore, Maryland), which detected an in-frame fusion between *exon 6 of FUS on chromosome 16 and exon 5 of CREB3L2 on chromosome 7,* confirming the results of the prior solid tumor fusion panel. Additionally, an *EWSR1* fusion was not detected.

At an 8-month postoperative assessment, clinical examination continued to show no evidence of disease or growth at the excision site, and positron emission tomography (PET)/CT scans, extending from the skull base to the mid-thigh, showed no active soft-tissue malignancy or organ metastasis. Conservative excisional surgical margins had been performed as the lesion was considered preoperatively as a benign pathologic process. Accordingly, the patient was advised to undergo adjuvant radiotherapy due to concerns for adequate tumor margins. At 10 months postoperatively, the patient began adjuvant intensity-modulated radiation therapy to the right floor of the mouth, delivered at 5000 cGy in 25 fractions over 5 weeks. 

A 1-year postoperative clinical follow-up assessment showed no evidence of disease, and the patient was asymptomatic. Plasma was subsequently collected for Signatera testing (Natera, Austin, Texas), a highly sensitive assay measuring circulating tumor DNA (ctDNA) to assess for molecular residual disease and stratifying recurrence risk. Six tumor-informed assays were designed using the patient’s initial excision specimen. The plasma was analyzed with these assays, each targeting specific mutations identified in the patient’s tumor. The results of the Signatera test indicated no ctDNA was detected and the plasma was negative for all 6 target variants.

## Discussion

The detection of a submucosal swelling arising in the floor of the mouth should prompt clinicians to initially rule out any infectious or non-infectious odontogenic processes first. Then, other common pathoses within the clinical differential diagnosis of such a presentation would include salivary gland inflammatory lesions (ranula, sialadenitis, sialolithiasis), developmental entities (dermoid cyst, epidermoid cyst), or primary soft tissue neoplasms, such as benign (lipoma, rhabdomyoma, schwannoma) or malignant lesions (squamous cell carcinoma, mucoepidermoid carcinoma, adenoid cystic carcinoma, polymorphous adenocarcinoma, rhabdomyosarcoma).

Historically, LGFMS had been included in a group of hyalinizing spindle cell tumors (HSCT) with giant rosettes and an indolent growth in the early follow-up period [11]. This distinction was functionally erased by a comprehensive clinicopathologic study by Folpe et al., which reevaluated a large cohort of cases and demonstrated that LGFMS and HSCT were opposing ends of the same disease spectrum [5]. This conclusion was later conclusively proven by the identification of the shared *FUS::CREB3L2* chromosomal translocation *t(7;16)(q33;p11),* establishing them as a single biological diagnosis [12]. Longer-term follow-up assessments of LGFMS also revealed their significantly increased potential for metastasis [13, 14].

Another nosological confusion was the consideration that LGFMS represented a histomorphological variant of sclerosing epithelioid fibrosarcoma (SEF) [15]. While LGFMS is defined by the *FUS::CREB3L2* gene fusion and SEF by *EWSR::CREB3L1* fusion, the presence of some microscopic features alone was unsatisfactory, blurring the lines of distinction and confidence with classification. As such, a significant diagnostic challenge exists with many of the earlier published cases of LGFMS of the head and neck, as they lacked modern molecular or IHC supportive testing, and should be interpreted with caution. At present, SEF is characterized by its greater aggressive biology, older age of onset, increased tendency for metastasis, poorer prognosis, and varied gene expressions [9].

Histologically, LGFMS overlaps with a broad category of fibroblastic spindle cell neoplastic entities, characterized by bland spindle cells arranged in a whorled pattern within alternating collagenous and myxoid areas, and lesions may exhibit gross infiltration into surrounding tissues [16]. MUC4 has proven to be a sensitive IHC diagnostic tumor marker, in combination with the negativity of S100, desmin, caldesmon, cytokeratin, SMA and CD117, which aid in excluding other morphologically overlapping entities, including malignant peripheral nerve sheath tumor, nodular fasciitis, and other myxoid spindle cell tumors (such as myxoid neurofibroma and perineurioma) [6, 17].

Nonetheless, some LGFMS cases confirmed by *FUS::CREB3L2* gene fusion on molecular analysis have shown MUC4 negativity [18]. Consequently, the reported descriptive features overlapped considerably with several benign and low-grade malignant spindle cell lesions, particularly neurofibroma, fibromatosis, and low-grade myxofibrosarcoma. MUC4 immunoreactivity is highly sensitive for LGFMS but has also been reported in other neoplasms, such as glandular cell populations occurring in synovial sarcoma, malignant ossifying fibromyxoid tumor, and SEF, indicating that this biomarker and the overlapping spectrum of fusion variants are not entirely specific for LGFMS [6, 19–22]. Moreover, hybrid LGFMS/SEF tumors have been reported with overlapping EWSR1::CREB3L1 gene fusions and demonstrated positive MUC4 immunoexpression [20, 23].

Demographically, overall case series of LGFMS have shown a slight male predominance [14, 24, 25] or with near-equal sex predilection [7, 10, 26], with a mean age at diagnosis of 32.7 to 38.6 years (range: 2 to 98 years) [7, 9, 14, 25]. The majority of tumors have been found in the deep soft tissue sites of the lower extremities, trunk wall, and viscera [7]. Within the head and neck, most cases of LGFMS have been located with the soft tissues of the neck, with fewer lesions involving the face, ear, oropharynx, tongue, salivary glands, palate, maxillary/ethmoid sinuses, and buccal/labial mucosa; intraosseous sites have mainly involved the jaws and less often with orbit and intracranial extension [7, 27, 28]. The diagnosis of LGFMS is rare and typically relies on correlation between CT/magnetic resonance imaging and histopathological examination, and confirmed by molecular analysis.

Despite its bland microscopic appearance, long-term follow-up evaluations of LGFMS have been reported with rates of recurrence of 25.8 to 66.6% (with median age at diagnosis of 3.2 to 3.5 years) and risk of distant metastasis of 18.5% [2, 7, 14]. Isolated cases of LGFMS have metastasized from 45 to 50 years from initial pathologic diagnosis [2, 5]. Sites of metastatic spread of LGFMS have mainly involved the lung, pleura, and bone [2, 5]. Thus, the significant risks of late recurrence and metastasis potential emphasize the need for indefinite surveillance [7, 29]. The primary modality of treatment has involved wide en bloc surgical resection, although radiotherapy or chemotherapy has been utilized in severe or recurrent cases [7, 26]. Incident death associated with LGFMS has varied widely, from 3.2% of (6/186) to 42.4% (14/33) of patients with follow-up periods ranging from 3 months to 50 years [5, 14].

Our literature search yielded only 22 cases of LGFMS (including our featured case), within the head and neck that included genetic results, limiting rigorous clinicopathologic analysis; 18 of these published cases included treatment outcomes, and have been summarized in Table 1 [2, 6, 7, 12, 24–26, 30–37]. None of the affected patients died of disease. All patients were initially managed with surgically (biopsy and/or excision), with margins ranging from < 1 mm to 1 cm. Sixty-percent of patients (15/22) remained without evidence of disease at last follow up (ranging from 1 month to 21 years). However, 4 patients developed tumor recurrence, resulting in a recurrence rate of 22.2%, ranging from 3 to 156 months [2, 25, 30]. Notably, 2 patients had minimal or uncertain tumor margins, 1 of whom experienced recurrence at 156 months and developed lung metastasis [30]. One case of LGFMS of the mandible underwent transformation to SEF at 20 years (with unstated outcome) [35]. Three cases underwent surgery (1 each: biopsy, en bloc resection, excisional biopsy) and received supplemental radiotherapy, resulting in 1 patient with recurrence at 3 months, 1 alive with disease at 48 months, and our patient, who had prophylactic radiotherapy due to concerns about adequate tumor margins) and demonstrated no evidence at 1 year [25, 26]. Giani et al. proposed that affected patients with incomplete resection and minimal tumor-free margins will likely benefit with follow-up radiotherapy [29].

**Table 1:** Reported cases of low-grade fibromyxoid sarcoma with molecular findings in the head and neck region

Case #	Age (yrs)/sex	Site	Molecular finding/genetic modality	Treatment	Outcome	References^#^
1	35/M	Supraclavicular	t(7:16)(q34;p11) FUS rearrangement	Marginal excision	NED-1 mo	Reid et al. [12]
2	25/F	Neck	FUS::CREB3L2 fusion exon 7, CREB3L2 exon 5 (PCR)	Excision	Recurrence and lung metastasis-156 mos NED-21 yrs	Mertens et al. [30]
3	22/M	Neck	FUS::CREB3L2 fusion exon 7, CREB3L2 exon 5 (PCR)	Excision	NED-38 mos	Guillou et al. [6]
4	26/F	Brain	FUS rearrangement (FISH)	Complete excision	NED-5 yrs	Chen et al. [31]
5	5/F	Intracranial	FUS::CREB3L2 fusion	Complete excision	NED-18 mos	White et al. [32]
6	16/M	Maxilla	FUS rearrangement (FISH)	Resection	NED-6 mos	Spalthoff et al. [33]
7	31/F	Neck	FUS rearrangement (FISH)	Excision	NS	Vallejo-Benítez et al. [34]
8	28/M	Neck	FUS rearrangement (FISH)	Excision-negative margins	LTF	Li et al. [8]
9	22/F	Neck	FUS rearrangement (FISH)	Excision-negative margins	LTF	Li et al. [8]
10	55/F	Mandible	EWSR1::CREB3L2 fusion (NGS)	Segmental resection	Transformation to SEF-20 yrs, outcome NS	Laliberte et al. [35]
11	17/M	Tongue	FUS::CREB3L2 fusion	Biopsy, follow-up resection with 1 cm margins	NED-6 mos	Pellini et al. [36]
12	5/F	Neck	FUS rearrangement (FISH)	Resection with < 1 mm margins	Local recurrence, NED-16 mos	Scheer et al. [2]
13	16/M	Maxilla	FUS rearrangement (FISH)	Resection with unclear margins	Local recurrence, NED-7 mos	Scheer et al. [2]
14	11/M	Mandible	FUS rearrangement (FISH)	Partial resection with microscopic margins, reresection	NED-31 mos	Scheer et al. [2]
15	28/M	Neck	FUS rearrangement (FISH)	Excision	NED-36 mos	Gjorgova Gjeorgjievski et al. [26]
16	28/F	Neck	FUS rearrangement (FISH)	Excision	NED-36 mos	Gjorgova Gjeorgjievski et al. [26]
17	97/F	Supraclavicular	FUS rearrangement (FISH)	Biopsy, local radiotherapy	AWD-48 mos	Gjorgova Gjeorgjievski et al. [26]
18	3/M	Lower lip	FUS rearrangement (FISH)	Excision	NS	Gjorgova Gjeorgjievski et al. [26]
19	22/F	Parotid gland	EWSR1::CREB3L2 fusion (NGS)	Excision	NED-3 yrs	Alayed and Pharaon [24]
20	56/M	Maxillary sinus	FUS rearrangement (FISH)	En-block resection, radiotherapy	Recurrence-3 mos. Outcome NS	Doblan [25]
21	70/M	Orbit	EWSR1::CREB3L2 fusion (NGS)	Excision with orbitotomy and marginotomy	NED-4 mos, persistent diplopia-9 mos	Oh et al. [37]
22	18/M	Floor of mouth	FUS::CREB3L2 fusion exon 6, CREB3L2 exon 5 (STFP)	Complete resection, radiotherapy-5000 cGy (25 fractions)	NED-12 mos	Present case

^#^, number; yrs, years; M, male; NED, no evidence of disease; mos, months; PCR, polymerase chain reaction; F, female; FISH, fluorescence in situ hybridization; LTF, lost to follow-up; SEF, sclerosing epithelioid fibrosarcoma; NGS, next generation sequencing; AWD, alive with disease; NS, not specified; STFP, solid tumor fusion panel

To our knowledge, the presented case is the first published account of LGFMS arising in the floor of the mouth and confirmed with *FUS::CREB3L2* fusion. At a 1-year postoperative assessment, Signatera testing indicated no detectable tumor ctDNA. It remains unclear whether the negative result with Signatera at the 1-year follow-up offered any long-term prognostic benefit. A recent preliminary study of various soft tissue sarcomas showed that use of this diagnostic modality offered comparable measures of tumor progression with radiologic imaging [38]. In addition, other investigations have corroborated its prognostic utility, such as with undifferentiated pleomorphic sarcoma [39], uterine malignancies [40], and a diversity of pediatric solid tumors [41]. As far as could be ascertained, this article represents the first published case report on the employment of the Signatera assay for postoperative surveillance of LGFMS of the head and neck.

There are several limitations with this report, principally with the clinical utility of a novel case and the short-term (1-year) postoperative follow-up. Furthermore, the prognostic applicability of the molecular analysis and postoperative biomarker assay should be viewed with caution in the context of single cases. It is advocated that more cases of LGFMS of the head and neck are published that contain enhanced diagnostic and prognostic techniques.

## Conclusion

This report confirms the novel clinical presentation of LGFMS of the floor of the mouth, underscoring the variability of tumor site. Positive immunostaining with MUC4 narrowed the list of low-grade spindle cell neoplasms. RNA sequencing detected *FUS::CREB3L2* fusion and confirmed the diagnosis of LGFMS, underscoring the critical role of molecular analysis in distinguishing this malignancy from its benign mimics. Despite the rarity of LGFMS, it should be included within the differential diagnosis of floor of the mouth swellings that reveal a histological profile of bland spindle cell proliferation within a myxomatous stroma.

## Supplementary Information

Below is the link to the electronic supplementary material.

Supplementary Material 1

## Data Availability

Access to data will be available by request to the corresponding author.
