# Correction: Novel Case of a Molecularly Confirmed Low-Grade Fibromyxoid Sarcoma of the Floor of the Mouth and Literature Review

**DOI:** 10.1007/s12105-026-01906-2

**Published:** 2026-05-29

**Authors:** Eugene G. Bestman, John K. Brooks, Scott D. Nelson, Ahmed S. Sultan, Njood Hawari, Samantha L. Jeffrey, Jettie Uyanne, Felix Kyle Yip, Kevin Artis, Manando Nakasaki

**Affiliations:** 1 Department of Oral and Maxillofacial Surgery, Harbor-UCLA Medical Center, 1000 W Carson Street, Torrance, Los Angeles, CA 90502 USA; 2 Department of Oncology and Diagnostic Sciences, University of Maryland School of Dentistry, Baltimore, MD USA; 3 Department of Pathology and Laboratory Medicine, David Geffen School of Medicine, University of California, Los Angeles, CA USA; 4 University of Maryland Marlene and Stewart Greenebaum Comprehensive Cancer Center, Baltimore, MD USA; 5 Department of Oncology and Diagnostic Sciences, Oral and Maxillofacial Pathology, University of Maryland School of Dentistry, Baltimore, MD USA; 6 Sarcoma Oncology Research Center, Cancer Center of Southern California, Santa Monica, CA USA

**Correction to: Head and Neck Pathology (2026) 20:18** 10.1007/s12105-026-01888-1

In the original version of this published article, Figs. 3a and b were superimposed on Figs. 4a and b, resulting in incorrect figure presentation in the pdf version. Their presentation has now been corrected. The original article has been corrected.

**Fig. 3:**
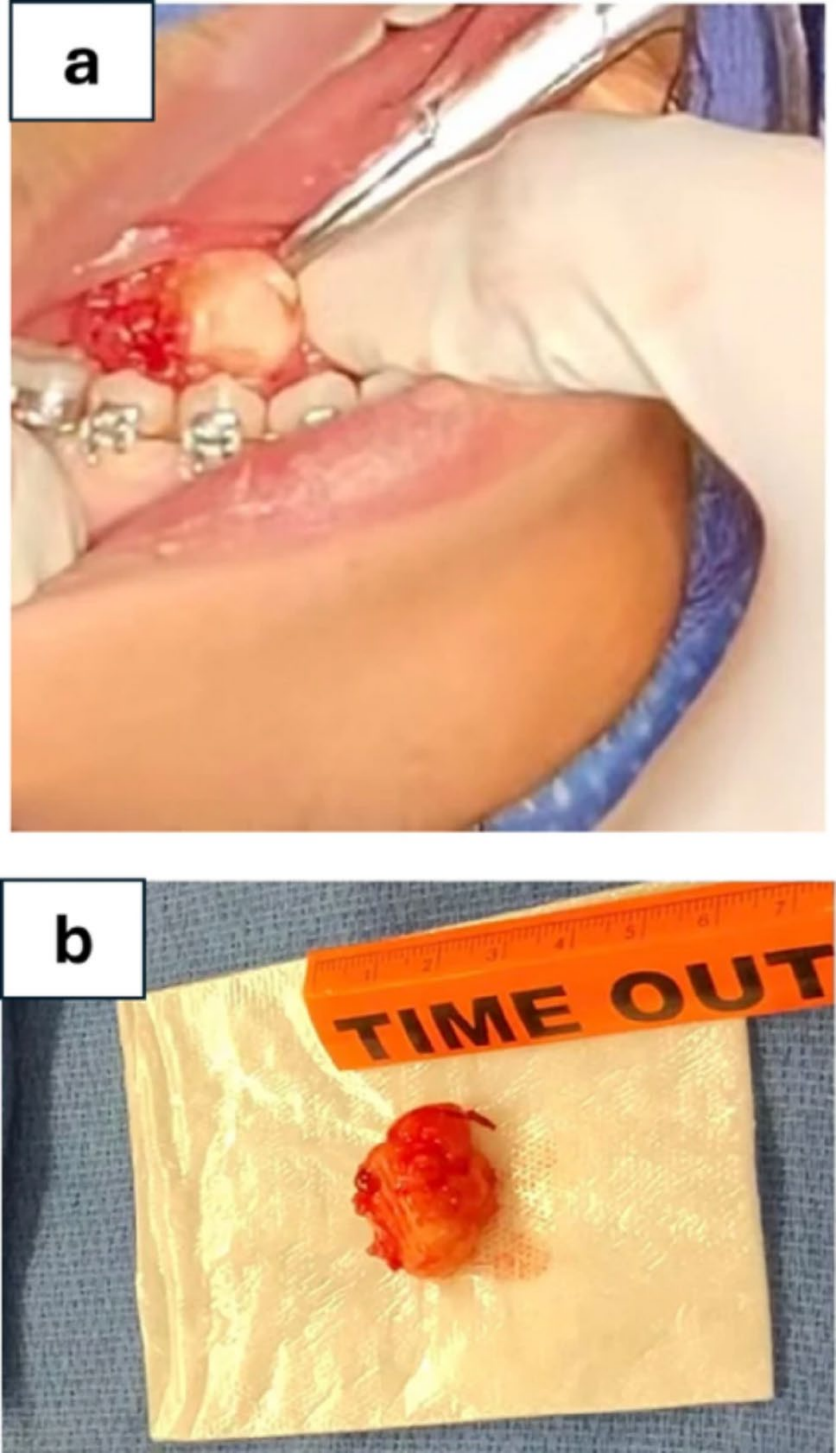
Operative views. **a** Capsulated lesion. **b** Surgical specimen

**Fig. 4:**
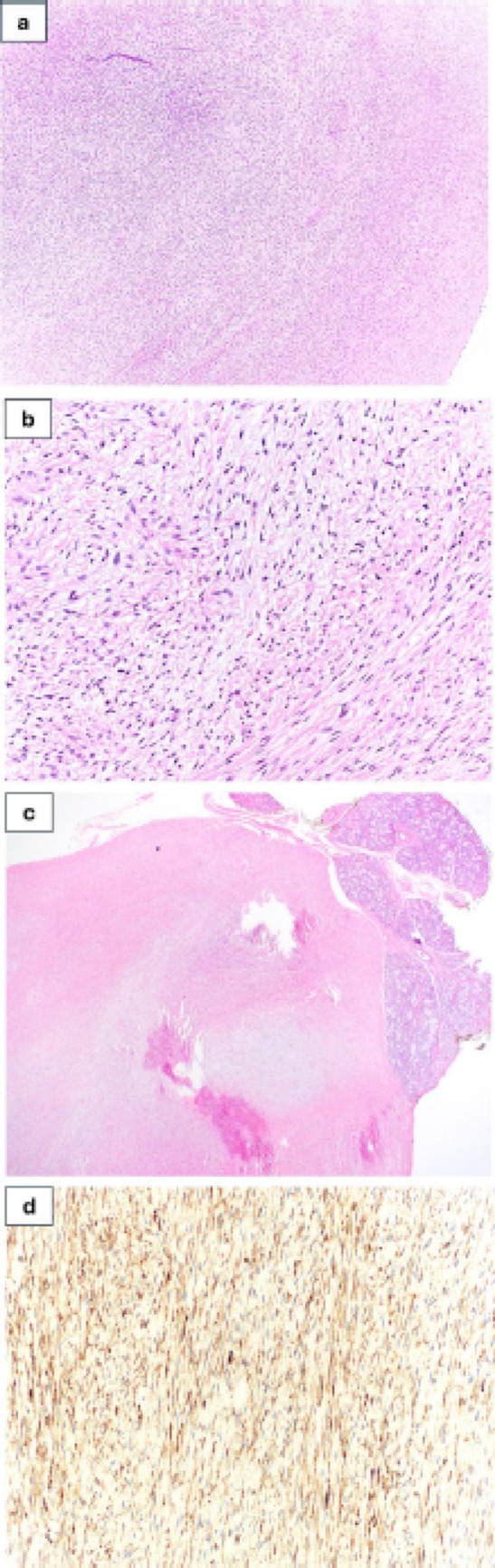
Photomicrographs show representative findings of LGFMS. **a** Moderately cellular spindle cell proliferation arranged in short fascicles within a collagenous stroma containing fine curvilinear blood vessels and focal myxoid transitional areas (hematoxylin and eosin staining, original magnification 40x). **b** Bland appearing spindle cells with mild nuclear pleomorphism (hematoxylin and eosin staining, original magnification 200×). **c** Focal areas of normal salivary glands adjacent to the tumor (hematoxylin and eosin staining, original magnification 20×). **d** Immunohistochemical staining was diffusely positive for MUC4 in tumor cells (original magnification 200×)

