# Supplementary material for: Novel Case of a Molecularly Confirmed Low-Grade Fibromyxoid Sarcoma of the Floor of the Mouth and Literature Review
Source: Head Neck Pathol. 2026 Jan 30;20(1):18. doi: 10.1007/s12105-026-01888-1 (PMC12858700; doi:10.1007/s12105-026-01888-1)
Supplement: Supplementary file 1 — Supplementary Material 1 [file 12105_2026_1888_MOESM1_ESM.docx]

**SUPPLEMENT. LIST OF REFERENCES OF 129 CASES OF LOW-GRADE FIBROMYXOID SARCOMA OF THE HEAD AND NECK**

**Note: Listed articles do not align with the reference numbers cited within the manuscript. Many of these references contain more than one case of low-grade fibromyxoid sarcoma.**

1. Devaney DM, Dervan P, O'Neill S, Carney D, Leader M (1990) Low-grade fibromyxoid sarcoma. Histopathology 17(5):463-465. https://doi.org/10.1111/j.1365-2559.1990.tb00769.x
2. Paulus W, Slowik F, Jellinger K (1991) Primary intracranial sarcomas: histopathological features of 19 cases. Histopathology 18(5):395-402. https://doi.org/10.1111/j.1365-2559.1991.tb00869.x
3. Evans H (1993) Low-grade fibromyxoid sarcoma. A report of 12 cases. Am J Surg Pathol 17(6):595-600. https://doi.org/10.1097/00000478-199306000-00007
4. Lane KL, Shannon RJ, Weiss SW (1997) Hyalinizing spindle cell tumor with giant rosettes: a distinctive tumor closely resembling low-grade fibromyxoid sarcoma. Am J Surg Pathol 21(12):1481-1488. https://doi.org/10.1097/00000478-199712000-00011
5. Papadimitriou JC, Ord RA, Drachenberg CB (1997) Head and neck fibromyxoid sarcoma: clinicopathological correlation with emphasis on peculiar ultrastructural features related to collagen processing. Ultrastruct Pathol 21(1):81-87. https://doi.org/10.3109/01913129709023250
6. Folpe AL, Lane KL, Paull G, Weiss SW (2000) Low-grade fibromyxoid sarcoma and hyalinizing spindle cell tumor with giant rosettes: a clinicopathologic study of 73 cases supporting their identity and assessing the impact of high-grade areas. Am J Surg Pathol 24(10):1353-1360. https://doi.org/10.1097/00000478-200010000-00004
7. Zámecník M, Michal M (2000) Low-grade fibromyxoid sarcoma: a report of eight cases with histologic, immunohistochemical, and ultrastructural study. Ann Diagn Pathol 4(4):207-217. https://doi.org/10.1053/adpa.2000.8122
8. Reid R, de Silva MV, Paterson L, Ryan E, Fisher C (2003) Low-grade fibromyxoid sarcoma and hyalinizing spindle cell tumor with giant rosettes share a common t(7;16)(q34;p11) translocation. Am J Surg Pathol 27(9):1229-1236. https://doi.org/10.1097/00000478-200309000-00006
9. Oda Y, Takahira T, Kawaguchi K, et al (2004) Low-grade fibromyxoid sarcoma versus low-grade myxofibrosarcoma in the extremities and trunk. A comparison of clinicopathological and immunohistochemical features. Histopathology 45(1):29-38. https//doi.org/10.1111/j.1365-2559.2004.01886.x
10. Antonescu CR, Baren A (2004) Spectrum of low-grade fibrosarcomas: a comparative ultrastructural analysis of low-grade myxofibrosarcoma and fibromyxoid sarcoma. Ultrastruct Pathol 28(5-6):321-332. https//doi.org/10.1080/019131290882259
11. Mertens F, Antonescu CR, Hohenberger P, et al (2005) Clinicopathologic and molecular genetic characterization of low-grade fibromyxoid sarcoma, and cloning of a novel FUS/CREB3L1 fusion gene. Lab Invest 85(3):408-415. <https://doi.org/10.1038/labinvest.3700274>
12. Bhattacharya B, Dilworth HP, Iacobuzio-Donahue C, et al (2005) Nuclear beta-catenin expression distinguishes deep fibromatosis from other benign and malignant fibroblastic and myofibroblastic lesions. Am J Surg Pathol 29(5):653-9. https://doi.org/10.1097/01.pas.0000157938.95785.da
13. Botev B, Casale M, Vincenzi B, et al (2006) A giant sarcoma of the parotid gland: a case report and review of the literature. In Vivo 20(6B):907-910.
14. Guillou L, Benhattar J, Gengler C, et al (2007) Translocation-positive low-grade fibromyxoid sarcoma: clinicopathologic and molecular analysis of a series expanding the morphologic spectrum and suggesting potential relationship to sclerosing epithelioid fibrosarcoma. Am J Surg Pathol 31(9):1387-1402. https://doi.org/10.1097/PAS.0b013e3180321959
15. Marglani O, Commons S, Lamothe A (2007) Radiation-induced low-grade fibromyxoid sarcoma of the sternocleidomastoid muscle. J Otolaryngol 36(5):E73-E75.
16. Tun K, Ozen O, Kaptanoglu E, Gurcan O, Beskonakli E, Celasun B (2008) Primary intracranial low-grade fibromyxoid sarcoma (Evans tumor). J Clin Neurosci 15(11):1298-1301. https://doi.org/10.1016/j.jocn.2007.07.085
17. Saito R, Kumabe T, Watanabe M, et al (2008) Low-grade fibromyxoid sarcoma of intracranial origin. J Neurosurg 108(4):798-802. https://doi.org/10.3171/JNS/2008/108/4/0798
18. Wu X, Petrovic V, Torode IP, Chow CW (2009) Low grade fibromyxoid sarcoma: problems in the diagnosis and management of a malignant tumour with bland histological appearance. Pathology 41(2):155-160. https://doi.org/10.1080/00313020802579276
19. Merchant SH (2009) Low grade fibromyxoid sarcoma: report of a case with epithelioid cell morphology, masquerading as a papillary thyroid carcinoma. Acta Cytol 53(6):689-692. https://doi.org/10.1159/000325411
20. Meng GZ, Zhang HY, Bu H, Geng JG (2009) Low-grade fibromyxoid sarcoma versus fibromatosis: a comparative study of clinicopathological and immunohistochemical features. Diagn Cytopathol 37(2):96-102. https://doi.org/10.1002/dc.20967
21. Tang Z, Zhou ZH, Lv CT, Qin LY, Wang Y, Tian G, et al (2010) Low-grade fibromyxoid sarcoma: clinical study and case report. J Oral Maxillofac Surg 68(4):873-884. <https://doi.org/10.1016/j.joms.2009.04.136>
22. Rekhi B, Deshmukh M, Jambhekar NA (2011) Low-grade fibromyxoid sarcoma: a clinicopathologic study of 18 cases, including histopathologic relationship with sclerosing epithelioid fibrosarcoma in a subset of cases. Ann Diagn Pathol 15(5):303-311. https://doi.org/10.1016/j.anndiagpath.2011.02.005
23. Evans HL (2011) Low-grade fibromyxoid sarcoma: a clinicopathologic study of 33 cases with long-term follow-up. Am J Surg Pathol 35(10):1450-1462. https://doi.org/10.107/PAS.0b013e31822b3687
24. Manes RP, Lemeshev Y, Batra PS (2011) Pathology quiz case 2. Low-grade fibromyxoid sarcoma (LGFMS). Arch Otolaryngol Head Neck Surg 137(2):199, 201-202. https://doi.org/10.1001/archoto.2010.248-a
25. Abe Y, Hashimoto I, Nakanishi H (2012) Recurring facial low-grade fibromyxoid sarcoma in an elderly patient: a case report. J Med Invest 59(3-4):266-269. https://doi.org/10.2152/jmi.59.266
26. Hwang S, Kelliher E, Hameed M (2012) Imaging features of low-grade fibromyxoid sarcoma (Evans tumor). Skeletal Radiol 41(10):1263-1272. https://doi.org/10.1007/s00256-012-1417-2
27. He KF, Jia J, Zhao YF (2013) Low-grade fibromyxoid sarcoma with cystic appearance and osseous metaplasia in the cheek: a case report and review of the literature. J Oral Maxillofac Surg 71(6):1143-1150. https://doi.org/10.1016/j.joms.2012.12.017
28. Maretty-Nielsen K, Baerentzen S, Keller J, Dyrop HB, Safwat A (2013) Low-grade fibromyxoid sarcoma: incidence, treatment strategy of metastases, and clinical significance of the FUS gene. Sarcoma 2013:256280. <https://doi.org/10.1155/2013/256280>
29. Dong W, Zhang H (2013) Low-grade fibromyxoid sarcoma of the thyroid: a case report. Ann Acad Med Singap 42(1):55-56.
30. Sargar K, Kao SC, Spunt SL, et al (2015) MRI and CT of low-grade fibromyxoid sarcoma in children: a report from Children's Oncology Group Study ARST0332. AJR Am J Roentgenol 205(2):414-420. https://doi.org/10.2214/AJR.14.13972
31. Lee EJ, Hwang HJ, Byeon HK, Park HS, Choi HS (2015) A low grade fibromyxoid sarcoma originating from the masseter muscle: a case report. J Med Case Rep 9:176. https://doi.org/10.1186/s13256-015-0658-9
32. Soma S, Bhat S, Shetty SK (2015) Low grade fibromyxoid sarcoma of the palate: a case report. J Clin Diagn Res 9(10):XD01-XD02. https://doi.org/10.7860/JCDR/2015/14670.6557
33. Mastoraki A, Strigkos T, Tatakis FP, Christophi A, Smyrniotis V (2015) Recurrent low-grade fibromyxoid sarcoma of the neck: report of a case and review of the literature. Indian J Surg Oncol 6(3):296-299. https://doi.org/10.1007/s13193-015-0429-5
34. Chen N, Gong J, Nie L, et al (2015) Primary intracranial low-grade fibromyxoid sarcoma with FUS gene rearrangement. Neuropathology 35(4):348-353. <https://doi.org/10.1111/neup.12197>
35. White IK, Scherer AG, Baumanis MM, Abdulkader M, Fulkerson DH (2015) Rapidly enlarging low-grade fibromyxoid sarcoma with intracranial extension in a 5-year-old girl: case report. J Neurosurg Pediatr 201516(4):372-376. <https://doi.org/10.3171/2015.3.PEDS14564>
36. Varsak YK, Arbag H, Yesildemir HS, Esen H (2015) Low-grade fibromyxoid sarcoma of superior turbinate in a pediatric patient. J Craniofac Surg 26(3):962-4. https://doi.org/10.1097/SCS.0000000000001481
37. Cowan ML, Thompson LD, Leon ME, Bishop JA (2016) Low-grade fibromyxoid sarcoma of the head and neck: a clinicopathologic series and review of the literature. Head Neck Pathol 10(2):161-166. https://doi.org/10.1007/s12105-015-0647-8
38. Chaudhuri K, Kasimsetty CR, Lingappa A, Gujjar PV (2016) Low-grade fibromyxoid sarcoma involving the mandible: a diagnostic dilemma. J Oral Maxillofac 20(2):334. https://doi.org/10.4103/0973-029X.185914
39. Spalthoff S, Bredt M, Gellrich NC, Jehn P (2016) A rare pathology: low-grade fibromyxoid sarcoma of the maxilla. J Oral Maxillofac Surg 74(1):219.e1-e10. <https://doi.org/10.1016/j.joms.2015.09.018>
40. Tatari MM, Elhariti L, Abou-elfadl M, et al (2016) Low grade fibromyxoid sarcoma of the neck: a case report. Ann Clin Case Rep 1:1056.
41. Lee JH, Choi HJ, Jung HY (2016) Low-grade fibromyxoid sarcoma of the malar area. Arch Plast Surg 43(1):110-112. https://doi.org/10.5999/aps.2016.43.1.110
42. Zakiyah R (2017) An infantile low grade fibromyxosarcoma of the neck: a rare case. J Islamic Med Res 1(2):74-81.
43. Vallejo-Benítez A, Rodríguez-Zarco E, Carrasco SP, et al (2017) Expression of dog1 in low-grade fibromyxoid sarcoma: a study of 19 cases and review of the literature. Ann Diagn Pathol 30:8-11. https://doi.org/10.1016/j.anndiagpath.2017.05.002
44. Laliberte C, Leong IT, Holmes H, Monteiro EA, O'Sullivan B, Dickson BC (2018) Sclerosing epithelioid fibrosarcoma of the jaw: late recurrence from a low grade fibromyxoid sarcoma. Head Neck Pathol 12(4):619-622. https://doi.org/10.1007/s12105-017-0879-x
45. Li M, Chen H, Shi D, et al (2018) Low-grade fibromyxoid sarcoma: a clinicopathologic and molecular study of 10 genetically confirmed cases. Int J Clin Exp Pathol 11(12):5860-5868.
46. Rao R, Honavar SG, Mulay K, Reddy VA (2019) Primary orbital low-grade fibromyxoid sarcoma–A case report. Indian J Ophthalmol 67(4):568-570.
47. Pellini P, De Virgilio A, Petruzzi G, et al (2019) Low-grade fibromyxoid sarcoma of the tongue: a rare nosological entity. Otorinolaringologia 69(3):188-191.

https://doi.org/10.23736/S0392-6621.18.02205-1

1. Kanato T, Kalyani S, Lailyang T, Santosh D, Rebecca T, Charai H (2019) Low grade fibromyxoid sarcoma in oral cavity: a rare case report. Indian J Otolaryngol Head Neck Surg 71(Suppl 1):25-26. https://doi.org/10.1007/s12070-015-0946-0
2. Chetverikova E, Kasenõmm P (2019) Low-grade fibromyxoid sarcoma of the lateral skull base: presentation of two cases. Case Rep Otolaryngol 2019:7917040. https://doi.org/10.1155/2019/7917040
3. Yadav M, Nagocha VB, Sharma D, Garg S, Thanvi S (2019) Intracranial extradural low-grade fibromyxoid sarcoma presenting as proptosis in a human immunodeficiency virus (HIV) positive patient. Int J 2(5):142-148.
4. Scheer M, Vokuhl C, Veit-Friedrich I, et al (2020) Low-grade fibromyxoid sarcoma: a report of the Cooperative Weichteilsarkom Studiengruppe (CWS). Pediatr Blood Cancer 67(2):e28009. https://doi.org 10.1002/pbc.28009
5. Kumari K, Thota R, Chaudhary HL, Sharma MC, Thakar A, Singh G (2020) Low-grade fibromyxoid sarcoma of the external auditory canal: a rare pathology and unusual location. Head Neck Pathol 14(1):276-282. https://doi.org/10.1007/s12105-019-01030-4
6. Park JM, Lim HR, Kim JH, Lee DH (2020) Giant low-grade fibromyxoid sarcoma in the neck. Korean J Otorhinolaryngol Head Neck Surg 63(7):432-436. <https://doi.org/10.3342/kjorl-hns.2019.00857>
7. Flores RM, Rojas BV, Gómez VM, Burgos CL (2020) Low-grade fibromyxoid saroma of the maxilla. Report of a case. Oral Surg Oral Med Oral Pathol Oral Radiol 129(1):e33.
8. Koucky V, Kalfert D, Kodetova Novakova D, Plzak J (2021) Low-grade fibromyxoid sarcoma of the maxillary sinus. Biomed Pap Med Fac Univ Palacky Olomouc Czech Repub 165(3):342-345. https://doi.org/10.5507/bp.2020.032
9. Toro C, Costa P, Vecchio GM, Magro G (2020) Low-grade fibromyxoid sarcoma of the parapharyngeal space: a case report and review of the literature. Oral Maxillofac Surg Cases 6(2):100152. https://doi.org/10.1016/j.omsc.2020.100152
10. Mustafa S, VandenBussche CJ, Ali SZ, Siddiqui MT, Wakely PE Jr (2020) Cytomorphologic findings of low-grade fibromyxoid sarcoma. J Am Soc Cytopathol 9(3):191-201. https://doi.org/10.1016/j.jasc.2020.01.006
11. Deewani MH, Danish MH, Awan MS, Ud Din N (2021) Low-grade fibromyxoid sarcoma of the parapharyngeal space: an unusual location. BMJ Case Rep 14(5):e237083. https://doi.org/10.1136/bcr-2020-237083
12. Chamberlain F, Engelmann B, Al-Muderis O, et al (2020) Low-grade fibromyxoid sarcoma: treatment outcomes and efficacy of chemotherapy. In Vivo 34(1):239-245. https://doi.org/10.21873/invivo.11766
13. Naik VG, Rai KK, Shivakumar HR (2021). Low-grade fibromyxoid sarcoma: a rare case report. Natl J Maxillofac Surg 12(2):271-275. https://doi.org/10.4103/njms.NJMS_54_15
14. Chivchibashi DL, Pavlov P, Tzaneva M, Sapundzhiev N, Davidov G (2021) Radiation-induced low grade fibromyxoid sarcoma of the larynx: a case report and literature review. Folia Med (Plovdiv) 63(3):433-437. https://doi.org/10.3897/folmed.53.e56150
15. Omokanye HK, Ogunkeyede AO, Nasir AA, Ibrahi KOO (2022) Low grade fibromyxoid sarcoma of the parotid in a 5-year-old child. Niger Med J 63(1):77-81. https://doi.org/10.60787/NMJ-63-1-107
16. Alayed DM, Pharaon MM (2022) Low-grade fibromyxoid sarcoma featuring an unusual *ewsr1-creb3l2* gene fusion: report of a rare case arising in the parotid gland. Avicenna J Med 12(2):87-92. <https://doi.org/10.1055/s-0042-1749611>
17. Gjorgova Gjeorgjievski S, Fritchie K, Thangaiah JJ, et al (2022) Head and neck low-grade fibromyxoid sarcoma: a clinicopathologic study of 15 cases. Head Neck Pathol 16(2):434-443. https://doi.org/10.1007/s12105-021-01380-y
18. Penafort PVM, de Pauli Paglioni M, Siqueira SAC, et al (2022) Low-grade fibromyxoid sarcoma in labial mucosa: report of a rare case in a pediatric patient. Oral Oncol 135:106243. https://doi.org/10.1016/j.oraloncology.2022.106243
19. Guo Y, Hao Y, Guan G (2022) Low-grade fibromyxoid sarcoma in the middle ear as a rare location: a case report. Transl Pediatr 11(6):1034-1039. https://doi.org/10.21037/tp-22-196
20. Doblan A (2022) Low-grade fibromyxoid sarcomas with the maxillary sinus localization: a case report and review of the literature. Indian J Otolaryngol Head Neck Surg 74(Suppl 2):1442-1449. https://doi.org/10.1007/s12070-021-02562-4
21. Tian K, Johnstone K, Lambie D, Frankel A (2022) Low-grade fibromyxoid sarcoma with high-grade features, a rare finding. ANZ J Surg 92(6):1519-1521. https://doi.org/10.1111/ans.17308
22. Ronen S, Ko JS, Rubin BP, et al (2023) Superficial low-grade fibromyxoid sarcoma. J Cutan Pathol 50(2):147-154. https://doi.org/10.1111/cup.14325
23. Anehosur V, Kumar N, Visweswaran A, Kumar K, Prabhu A (2023) Low-grade fibromyxoid sarcoma in the hard palate: a rare case report and review of the literature. J Maxillofac Oral Surg 22(4):1180-1185. https://doi.org/10.1007/s12663-023-01953-0
24. Leone L, Reale M, Buccoliero A, Guidi M, Trabalzini F (2023) Low-grade fibromyxoid sarcoma of the oral cavity in an infant: the youngest patient in the world: a case report. J Clin Images Med Case Rep 4(9):2617.
25. Tally H, Al-Janabi MAH, AlDwairy H, Al-Shehabi Z, Ibrahim M (2024) Low-grade fibromyxoid sarcoma in laryngopharynx: the first case report in the literature. J Surg Case Rep 2024(3):rjae141. https://doi.org/10.1093/jscr/rjae141
26. Nimavat SM, Hirani N, Khilnani AK, Warpe B, Malhotra KG, Jain NP (2024) Rare case of low-grade fibro-myxoid sarcoma presenting as a lateral neck swelling. Inter J Otorhinolaryngol Head Neck Surg 10(6):776-779. https://dx.doi.org/10.18203/issn.2454-5929.ijohns20243528
27. Oh AJ, Singh P, Pirakitikulr N, Roelofs K, Glasgow BJ, Rootman DB (2024) Low-grade fibromyxoid sarcoma of the orbit. Orbit 43(3):375-379. https://doi.org/10.1080/01676830.2022.2149820
28. Quiceno E, Soliman MAR, Khan A, et al (2024) Supraclavicular artery island flap for treatment of cervical wound defects and persistent cerebrospinal fluid leaks: a technical note and systematic review of the literature. World Neurosurg 185:e915-e925. https://doi.org/10.1016/j.wneu.2024.02.151
29. Park H, Banegas DW, Han SY, et al (2024) Primary palatal sarcoma exhibiting EWSR1::RORß fusion: a first case report and literature review. Oral Surg Oral Med Oral Pathol Oral Radiol 138(6):e113-e119. https://doi.org/10.1016/j.oooo.2024.06.018
30. Blay JY, Tlemsani C, Toulmonde M, et al (2024) Sclerosing epithelioid fibrosarcoma (SEF) versus low grade fibromyxoid sarcoma (LGFMS): presentation and outcome in the nationwide NETSARC+ series of 330 patients over 13 years. Eur J Cancer 196:113454. https://doi.org/10.1016/j.ejca.2023.113454
31. dos Santos Lima CR, de Oliveira Moreira VH, de Paula Coelho V, et al (2025) Oral low-grade fibromyxoid sarcoma: a case report. Oral Surg Oral Med Oral Pathol Oral Radiol 139(5):e1.
32. Agrawal S, Mishra S, Raviraj KU, Jha N, Kumar A, Madhupriya (2025) Low-grade fibromyxoid sarcoma of the maxilla: a diagnosis not to be missed. Indian J Pathol Microbiol 68(3):589-591. <https://doi.org/10.4103/ijpm.ijpm_957_23>
33. Zhao D, Dai J, Hu Y, Wang T (2025) A case report on the short-term recurrence of low-grade fibromyxoid sarcoma in the maxillary sinus. Ear Nose Throat J 104(6):346-350. https://doi.org/10.1177/01455613241276673
